# Supplementary material for: The Xanthophyll Carotenoid Lutein Reduces the Invasive Potential of Pseudomonas aeruginosa and Increases Its Susceptibility to Tobramycin
Source: Int J Mol Sci. 2022 Jun 28;23(13):7199. doi: 10.3390/ijms23137199 (PMC9266958; doi:10.3390/ijms23137199)
Supplement: Supplementary file 1 [file ijms-23-07199-s001.zip › Table S1-S2 20220531.pdf]

Table S1. Comparison of inhibition activities of lutein (Lut) and 3′-dehydrolutein (DHLut) towards PAO1

PAO1 Virulence	% of inhibition	
	Lut at 22 μM	DHLut at 22 μM
• Biofilm formation	**56 ± 2**	**57 ± 2**
• Motilities		
• Swarming	**40 ± 9**	**36 ± 3**
• Twitching	**34 ± 5**	**37 ± 5**
• Virulence factors production		
• Pyocyanin	**67 ± 4**	**66 ± 6**
• Elastase B	**19 ± 2**	*8 ± 3*
• Rhamnolipids	**62 ± 5**	**64 ± 5**
• QS-related genes expression		
• *lasB*	**60 ± 4**	**61 ± 3**
• *rhlA*	**62 ± 2**	**64 ± 4**
• *rhlI*	**60 ± 3**	**50 ± 7**
• *rhlR*	**64 ± 3**	**68 ± 8**
• *lasI*	**56 ± 3**	**53 ± 5**
• *lasR*	**60 ± 5**	**61 ± 6**
• *vfr*	**16 ± 3**	**12 ± 2**
• *gacA*	2 ± 3	5 ± 6
• QS-independent genes expression		
• *aceA*	1 ± 3	2 ± 4

Value in bold were tested significant compared to DMSO condition

Table S2: *Pseudomonas aeruginosa* strains and plasmids used in this study

Strains or plasmids	Relevant characteristics	References
Strains		
P. aeruginosa PAO1	Wild-type (strain PAO0001; http://www.pseudomonas.med.ecu.edu/)	
P. aeruginosa ΔPA1432	*P. aeruginosa* transposon mutant ID11174; *lasI*::IS*lacZ*/hah;Tet^R	(Jacobs et al., 2003)
P. aeruginosa ΔPA3476	*P. aeruginosa* transposon mutant ID32454; *rhlI*::IS*phoA*/hah;Tet^R	(Jacobs et al., 2003)
Plasmids		
pLP170	Broad-host-range *lacZ* transcriptional fusion vector containing an RNase III splice sequence positioned between the multiple cloning site and *lacZ*; Cb^r	(Pesci et al., 1997)
pPCS1001	pLP170-derivative containing P_{lasR}-*lacZ* transcriptional fusion	(Pesci et al., 1997)
pLPR1	pLP170-derivative containing P_{rhlI}-*lacZ* transcriptional fusion	(Van Delden and Iglewski, 1998)
pPCS1002	pLP170-derivative containing P_{rhlR}-*lacZ* transcriptional fusion	(Pesci et al., 1997)
pLP170_*gacA*	pLP170- derivative containing P_{gacA}-*lacZ* transcriptional fusion	(Rasamiravaka et al., 2015)
pLP170_*vfr*	pLP170- derivative containing P_{vfr}-*lacZ* transcriptional fusion	(Rasamiravaka et al., 2015)
pQF50	Broad-host-range promoter-less *lacZ* transcriptional fusion vector; Cb^r	(Ishida et al., 2007)
p◎01	pQF50-derivative containing P_{lasB}-*lacZ* transcriptional fusion	(Ishida et al., 2007)
p◎02	pQF50-derivative containing P_{rhlA}-*lacZ* transcriptional fusion	(Ishida et al., 2007)
p◎03	pQF50-derivative containing P_{lasI}-*lacZ* transcriptional fusion	(Ishida et al., 2007)
pTB4124	pQF50-derivative containing P_{aceA}-*lacZ* transcriptional fusion	(Kretzschmar et al., 2008)

Tcr,tetracycline resistance; Cbr carbenicillin resistance
