# Supplementary figures and images for: The Xanthophyll Carotenoid Lutein Reduces the Invasive Potential of Pseudomonas aeruginosa and Increases Its Susceptibility to Tobramycin
Source: Int J Mol Sci. 2022 Jun 28;23(13):7199. doi: 10.3390/ijms23137199 (PMC9266958; doi:10.3390/ijms23137199)

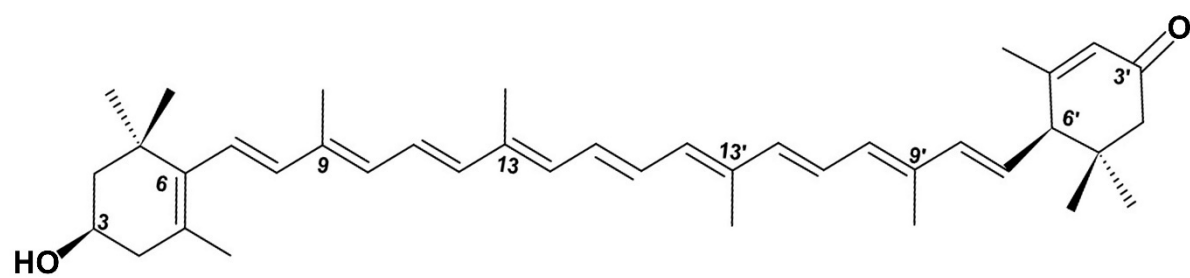

**Figure S1:** Chemical structure of dietary xanthophyll carotenoid 3'-dehydrolutein.

Supplement: Supplementary file 1 [file ijms-23-07199-s001.zip › Figure S1.pdf]
